# Sex Differences in All-Cause Inpatient Mortality Risk in Gastric Cancer: Nationwide Inpatient Population-Based Study

**DOI:** 10.7759/cureus.28602

**Published:** 2022-08-30

**Authors:** Sravani Kommuru, Yakub Ibrahim, Yash P Ashara, Karanpreet Singh, Maitri P Shah, Suchi D Shah, Trisha Saha

**Affiliations:** 1 Internal Medicine, Dr. Pinnamaneni Siddhartha Institute of Medical Sciences and Research Foundation, Vijayawada, IND; 2 Accident and Emergency, Mid and South Essex University Hospital NHS Trust, Southend On Sea, GBR; 3 Mayo Clinic Cancer Center, Mayo Clinic, Rochester, USA; 4 Medicine, Government Medical College and Hospital, Amritsar, USA; 5 Internal Medicine, Pandit Deendayal Upadhyay Medical College, Rajkot, IND; 6 Internal Medicine, Ahmedabad Municipal Corporation's Medical Education Trust Medical College, Ahmedabad, IND; 7 Internal Medicine, Columbia University College of Physicians and Surgeons, New York, USA

**Keywords:** gender, gastric cancer

## Abstract

Objective

The purpose of this study is to evaluate the differences in demographic characteristics, comorbidities, and hospital outcomes in gastric cancer inpatients by sex and evaluate the risk factors for in-hospital mortality in gastric cancer inpatients by sex.

Methods

We conducted a cross-sectional study using the nationwide inpatient sample (NIS, 2019). Our sample included 22,415 adult inpatients (age ≥18 years) hospitalized with a primary discharge diagnosis of gastric cancer that was identified by the international classification of diseases, 10th revision (ICD-10) codes of C16.x. Independent univariate binomial logistic regression models were used to evaluate the odds ratio (OR) of predictors associated with all-cause in-hospital mortality in gastric cancer inpatients by sex.

Results

The total number of patients admitted with gastric cancer was 22,415, out of which 62.7% were males and 37.3% were females, with the mean age at the admission of 65.5 years and 66.4 years, respectively. While studying comorbidities, we found that 41.5% percent of all patients had gastric cancer with metastasis, and there existed a significantly higher prevalence in males (42.2% vs. 40.4% in females). Other important and statistically significant comorbid conditions that were prevalent in these patients include complicated diabetes (12.2%), obesity (12.1%), depression (8%), and alcohol abuse (3.1%). Females between 50-59 years of age were at 2.5 times increased risk of mortality compared to those less than 40 years of age (OR: 2.5; 95% CI: 1.28-4.95).

Conclusion

Females of the age group 50-59 years are at greater risk of all-cause inpatient mortality due to gastric cancer. Black males are at increased risk of all-cause inpatient mortality compared to White males. Gastric cancer incidence and mortality rates have been down trending with the development of screening and better treatment options, but it still continues to be a major burden on the healthcare system.

## Introduction

There has been a significant decline in the prevalence of gastric cancer (GC), dropping by about 1.5% each year over the last decade, yet it contributes to the significant global cancer burden [1]. About one million new cases and approximately 783,000 deaths are recorded globally, ranking it as the third leading cause of cancer deaths worldwide [2]. In the United States (US), it represents 1.5% of all new cancer diagnoses, with reported incidence and mortality rates of 6.5 and 2.9 per 100,000 population, respectively [1,3]. Its incidence and prevalence vary in different regions of the world, being highest in East Asian countries compared to other regions, including North America [4,5]. Among its variants, adenocarcinoma is the most common histological type representing 90 to 95% of diagnosed gastric cancer [5].

GC is more common in males than females, and it is the fifth most common cancer worldwide, being the fourth most common cancer in men and the seventh most common cancer in women [6,7]. GC is 2.2 times more likely to be detected in males than females in developed countries, and the ratio is 1.8 in developing countries [4]. The incidence rates are higher in Latin America, Eastern and Central Asian countries, with an average incidence of 32.1 and 13.2 per 100,000 among males and females, respectively [6].

Gastric cancer has multifactorial pathogenesis involving genetic as well as environmental factors.* Helicobacter pylori (H. pylori)* and Epstein-Barr virus infections are well-known risk factors for GC [8]. Around 10% of cases are known to be genetic, and a greater percentage of cases are mostly sporadic in nature. The risk of developing GC with positive family history is three folds higher [7]. Lifestyle habits such as alcohol consumption and tobacco smoking are known to increase the risk of GC [8].

There are multiple studies in the literature exploring GC epidemiology, risk factors, and its treatment modalities. However, there is limited inpatient data delineating the epidemiological factors by gender. We would like to study the differences in demographic characteristics (including age, race/ethnicities, household income, and location) and comorbidities as well as hospital outcomes (including stay and cost, the severity of illness, in-hospital mortality, and utilization of procedures) in GC inpatients by sex. Additionally, we evaluate the risk factors for in-hospital mortality in GC inpatients by sex.

## Materials and methods

We conducted a cross-sectional cohort study using the nationwide inpatient sample (NIS, 2019). The NIS is the publicly available de-identified inpatient-based data that covers patient records from non-federal community hospitals across 48 states in the US. According to the agency for healthcare research and quality (AHRQ) and the department of health and human services, the utilization of the NIS does not require approval from an institutional review board [9]. We included 22,415 adult inpatients (age 18 years and above) hospitalized with a primary discharge diagnosis of gastric cancer. This was identified by extracting patient records with a primary diagnostic variable that included the international classification of diseases, 10th revision (ICD-10) codes of C16.x.

The variables of interest included demographic characteristics: age at admission, sex, race/ethnicity, and median household income (below/above the 50^th^ percentile). We included location as defined by US counties and developed by the national center for health statistics (NCHS). Metropolitan counties are defined as areas with 50,000 or more people, and micropolitan counties are areas with fewer than 50,000 people [9]. The comorbidities are the co-diagnoses in the patient records, and we included metastasis, an acquired immunodeficiency syndrome (AIDS), leukemia, lymphoma, dementia, diabetes (with and without chronic complications), hypertension, chronic pulmonary disease, peripheral vascular disease (PVD), obesity, alcohol abuse, depression, and drug abuse. Comorbid AIDS that is included in this study is the variable cm_AIDS as provided in the NIS, and that does not have clarification if it included patients with only human immunodeficiency virus (HIV) infection [9]. The hospitalization outcomes of interest included: severity of illness which was measured using the all-patient refined drugs (APR-DRGs) in the NIS, and the length of stay (LOS), total charges, and in-hospital mortality (all-cause) [9]. The treatment procedures identified in the patient records were gastrectomy, small bowel resection, duodenal resection, esophagectomy, and gastrostomy.

We used Pearson's chi-square test and descriptive statistics for categorical data and an independent-sample t-test for continuous data (age, LOS, and total charges) to measure the differences between gastric cancer inpatients by sex. Independent univariate binomial logistic regression models were used to evaluate the odds ratio (OR) of predictors associated with in-hospital mortality in gastric cancer inpatients for males, females, and the overall study sample. A p-value <0.05 was used to determine the statistical significance test result, and all analyses were conducted using the statistical package for the social sciences (SPSS) version 27 (IBM Corp., Armonk, USA).

## Results

The total number of patients admitted with a primary diagnosis of GC was 22,415, out of which 62.7% were males (n=13,880), and 37.3% were females (n=8,535), with the mean age at the admission of 65.5 years and 66.4 years, respectively. Elderly patients above the age of 70 made up a significant proportion (42.2%) of those hospitalized with GC. While comparing race/ethnicities, most of the patients were Whites (52.5%), followed by blacks (18.3%), Hispanics (16.4%), and others (12.9%). There existed a significant difference by sex, with a higher prevalence in Blacks and Hispanics in the female cohort. We found that most of these patients were from metropolitan counties (87.4%) and from low-income families with a median household income below the 50th percentile (51.7%).

While studying comorbidities, we found that 41.5% of all patients had GC with metastasis, and there existed a statistical difference in males (42.2% of males vs. 40.4% of females, p=0.007). Diabetes (13% vs. 10.8%, p<0.001) and alcohol abuse (4.4% vs. 1%, p<0.001) was more prevalent among the males whereas depression (6.5% vs. 10.4%, p<0.001) and obesity (10.8% vs. 14.2%, p<0.001) were more prevalent among the females.

Most of the patients underwent gastrectomy as a primary procedure (35.8%), followed by esophagectomy (3.9%) and gastrostomy (3.9%). There existed a non-significant difference in the mean LOS and cost during inpatient management of gastric cancer by sex. About 52.6% of the patients had a major loss of function, and the overall in-hospital mortality rate was 4.4%. The mortality rate was significantly higher in males (4.7%) compared to females (3.8%), as seen in Table 1.

**Table 1:** Differences in demographics and hospital outcomes in gastric cancer inpatients AIDS - acquired immunodeficiency syndrome; LOS - length of stay

Variable	Male	Female	Total	p-value
Number of inpatients	13,880	8,535	22,415	-
Mean age at admission, in years	65.5	66.4	-	<0.001
Age at admission, in %				
<40 years	3.6	4.8	4.1	<0.001
40-49 years	7.6	8.1	7.8	
50-59 years	18.9	15.8	17.7	
60-69 years	29.5	26.0	28.2	
≥70 years	40.3	45.3	42.2	
Race/ethnicity, in %				
White	56.4	46.1	52.5	<0.001
Black	17.0	20.3	18.3	
Hispanic	15.0	18.6	16.4	
Other	11.6	15.0	12.9	
Median household income				
below 50^th^ percentile	52.2	50.7	51.7	0.031
above 50^th^ percentile	47.8	49.3	48.3	
Location				
Metropolitan counties	85.8	90.0	87.4	<0.001
Micropolitan counties	8.0	5.4	7.0	
Other counties	6.2	4.6	5.6	
Comorbidities, in %				
Metastasis	42.2	40.4	41.5	0.007
AIDS	0.4	0.3	0.4	0.208
Leukemia	0.6	0.2	0.4	<0.001
Lymphoma	0.9	0.4	0.7	<0.001
Dementia	2.5	3.6	2.9	<0.001
Diabetes	13.0	10.8	12.2	<0.001
Hypertension	16.2	15.3	15.9	0.074
Chronic pulmonary disease	15.2	15.2	15.2	0.989
Peripheral vascular disease	6.2	5.5	5.9	0.044
Obesity	10.8	14.2	12.1	<0.001
Alcohol abuse	4.4	1.0	3.1	<0.001
Depression	6.5	10.4	8.0	<0.001
Drug abuse	1.3	1.0	1.2	0.025
Severity of illness, in %				
Minor loss of function	8.6	10.9	9.5	<0.001
Moderate loss of function	37.2	39.1	37.9	
Major loss of function	54.3	50.0	52.6	
Treatment procedures				
Gastrectomy	33.9	39.0	35.8	<0.001
Small bowel resection	1.4	1.6	1.5	0.158
Duodenal resection	1.2	1.5	1.3	0.043
Esophagectomy	4.9	2.2	3.9	<0.001
Gastrostomy	4.4	3.0	3.9	<0.001
Other outcomes				
Mean LOS, in days	7.7	7.8	-	0.162
Mean total charges, in $	108,349	104,902	-	0.043
In-hospital mortality, in %	4.7	3.8	4.4	0.001

We found that certain factors increase the risk of mortality in GC inpatients. Females between 50-59 years of age had 2.5 times increased risk of mortality compared to those less than 40 years of age (OR: 2.5; 95% CI: 1.28-4.95). However, we found that age was not a significant predictor of in-hospital mortality in the overall sample. The risk of in-hospital mortality was higher amongst Blacks compared to Whites (OR: 1.48; 95% CI: 1.24-1.76), more so for Black males (OR: 1.91; 95% CI: 1.55-2.37). Patients in micropolitan counties have an increased risk of mortality compared to those from metropolitan counties (OR: 1.58; 95% CI: 1.24-1.99). The odds of mortality among females admitted in micropolitan counties were noted to be 78% higher compared to females in metropolitan counties (OR: 1.78; 95% CI: 1.17-2.71).

While studying chronic comorbidities, we found that GC inpatients with metastasis and comorbid AIDS had an increased risk of mortality by two to three times. Metastasis increased the odds for in-hospital mortality in females (OR: 3.09; 95% CI: 2.60-3.68) and males (OR: 3.42; 95% CI: 2.68-4.37). When comorbid AIDS increased the risk only in males by three times (OR: 3.14; 95% CI: 1.50-6.55), then lymphoma increased mortality risk only among females (OR: 3.89; 95% CI: 1.34-11.38). Hypertension was found to increase mortality in GC patients, particularly males (OR: 1.56; 95% CI: 1.25-1.95), as shown in Table 2.

**Table 2:** Risk factors for in-hospital mortality in gastric cancer inpatients AIDS - acquired immunodeficiency syndrome; OR - odds ratio; CI - confidence interval

Variable	Male, OR (95% CI)	Female, OR (95% CI)	Overall total, OR (95% CI)
Age at admission, in %			
<40 years	Reference	Reference	Reference
40-49 years	0.74 (0.45-1.19)	1.29 (0.59-2.81)	0.91 (0.61-1.37)
50-59 years	0.80 (0.52-1.23)	2.52 (1.28-4.95)	1.21 (0.84-1.72)
60-69 years	0.75 (0.49-1.13)	1.94 (0.99-3.81)	1.06 (0.75-1.51)
≥70 years	0.98 (0.65-1.47)	1.41 (0.72-2.75)	1.12 (0.79-1.59)
Race/ethnicity			
White	Reference	Reference	Reference
Black	1.91 (1.55-2.37)	0.92 (0.67-1.25)	1.48 (1.24-1.76)
Hispanic	0.93 (0.72-1.21)	1.05 (0.77-1.42)	1.02 (0.84-1.24)
Other	1.21 (0.92-1.58)	0.46 (0.29-0.72)	0.88 (0.70-1.11)
Median household income			
below 50^th^ percentile	Reference	Reference	Reference
above 50^th^ percentile	1.16 (0.97-1.39)	0.79 (0.62-1.02)	1.02 (0.88-1.18)
Location			
Metropolitan counties	Reference	Reference	Reference
Micropolitan counties	1.55 (1.16-2.06)	1.78 (1.17-2.71)	1.58 (1.24-1.99)
Other counties	0.99 (0.67-1.47)	1.49 (0.91-2.45)	1.13 (0.83-1.54)
Comorbidities, in %			
Metastasis	3.09 (2.60-3.68)	3.42 (2.68-4.37)	3.16 (2.75-3.64)
AIDS	3.14 (1.50-6.55)	<0.001	2.47 (1.23-4.97)
Leukemia	1.33 (0.52-3.42)	<0.001	1.02 (0.40-2.59)
Lymphoma	0.67 (0.26-1.73)	3.89 (1.34-11.38)	1.29 (0.67-2.53)
Dementia	0.47 (0.24-0.89)	1.16 (0.59-2.25)	0.67 (0.42-1.07)
Diabetes	1.02 (0.79-1.31)	1.09 (0.88-1.35)	1.09 (0.88-1.35)
Hypertension	1.56 (1.25-1.95)	0.77 (0.53-1.12)	1.26 (1.05-1.53)
Chronic pulmonary disease	0.95 (0.75-1.21)	1.19 (0.87-1.63)	1.04 (0.87-1.26)
Peripheral vascular disease	1.17 (0.85-1.61)	1.46 (0.93-2.29)	1.29 (0.99-1.66)
Obesity	0.72 (0.53-0.97)	0.60 (0.41-0.89)	0.68 (0.54-0.87)
Alcohol abuse	0.56 (0.33-0.94)	<0.001	0.47 (0.28-0.79)
Depression	0.98 (0.70-1.38)	0.95 (0.64-1.41)	0.94 (0.73-1.21)
Drug abuse	<0.001	1.84 (0.72-4.74)	0.39 (0.16-0.95)

## Discussion

This cross-sectional study of GC inpatients revealed that overall mortality among inpatients with GC was 4.4%, with a higher mortality rate among males when compared to females (4.7% vs. 3.8%). Trending with past studies, we found incidence and mortality of gastric cancer were higher among males when compared to females [4,10]. Karimi et al. demonstrated that the incidence of GC increases with advancing age [11]. Along similar lines, this study demonstrated that a higher proportion (42.2%) of GC patients were of 70 years or older, this being true for both men and women. In evaluating the racial differences in GC mortality, the national institute of health (NIH) found that the mortality rate was highest in Black men (7.5 in 100,000) [10]. Our analysis revealed that Black males have approximately two times greater risk for mortality due to GC as compared to White men.

We found that diabetes was a significantly prevalent comorbid condition in gastric cancer patients among both men and women, with a slightly higher prevalence among the males (13% in men as opposed to 10.8% in women), but we also found that diabetes does not increase mortality in GC patients in both men and women. A 2017 meta-analysis stated that gender difference was not significantly associated with the relationship between diabetes and GC incidence (p=0.495) and gastric mortality (p=0.848) [11,12].

Documented literature suggests that 37% of GC cases are identified only after cancer has metastasized [10]. In the present analysis, we found that 41.5% of patients with GC had metastasis during the time of admission. We found that metastasis was prevalent among men and women and, therefore, it can't be classified as a sex-dependent risk factor. Contrary to this, some studies state that estrogen in females might be responsible for the progression of GC and metastasis, as in breast cancer [13]. Our study also indicated that metastasis increased the odds of mortality in both men and women by nearly three times, with a relatively higher risk in females. This finding is in line with existing literature on five-year survival rates in GC patients, which showed better five-year survival rates of up to 71.8% in localized GC as opposed to a 5.9% survival rate in GC patients with distant metastasis [10]. An important issue emerging from these findings is the implementation of screening procedures in high-risk populations. 

Our study revealed that obesity was significantly prevalent among gastric cancer patients, both men (10.8%) and women (14.2%), with a relatively higher prevalence in women. Existing literature confirms obesity as a risk factor for gastric cancer. Obesity can increase the risk of GC via several possible mechanisms. There is an increased prevalence of *Helicobacter pylori* infection in obese patients providing a clue to the increased incidence of gastric cancer in obese populations [14]. Obesity can promote gastroesophageal reflux disease, which predisposes Barrett's esophagus, a metaplastic precursor to gastric adenocarcinoma [15]. A Swedish study on the association between body mass and adenocarcinoma of the esophagus and gastric cardia found that the odds ratio for patients with cardia adenocarcinoma was 2.3 in those with the highest body mass index (BMI) quartile compared with those in the lowest BMI quartile and 4.3 among obese persons [16].

A very small proportion (0.4%) of our study population was found to have AIDS during the time of diagnosis. Nagata et al., in their cohort study on non-AIDS defining cancers, which included 1,001 AIDS patients, followed over an average of nine years, stated that GC was the third most common non-AIDS defining cancer next to liver and colon cancer. About 49% of patients had advanced tumor stage at diagnosis (stage III or IV), which can provide a possible explanation for the observed risk of increased mortality by three times in males with comorbid AIDS [17]. Another important finding was that co-existing lymphoma increased the risk of mortality in females with GC by nearly four times. To our knowledge, there is no existing literature on possible mechanisms that could be responsible for the possible increase in mortality.

Cancer patients and survivors suffer from psychological problems, such as depression which may interfere with the patient's ability to cope with the burden of the illness; it may decrease compliance, extend hospitalization, reduce the quality of life, and increase suicide risk [17]. Our study revealed that 6.5% of males and 10.4% of females with GC had co-diagnoses of depression during the time of admission. In a recent meta-analysis, a similar finding was noted where depression was much more prevalent in female GC patients [18]. Although our study did not reveal any correlation between depression and GC mortality, it is important to screen all patients for depression as it interferes with the quality of life.

Gastrectomy was the primary treatment procedure in 35.8% of patients with GC, and a significant proportion of patients developed moderate (37.9%) to severe (52.6%) loss of function following treatment. Gastrectomy is an invasive procedure with both acute and long-term post-procedure complications [19], but it is the definitive treatment for GC with nodal metastasis. Currently, there is no recommended routine screening for GC in the US; a high index of clinical suspicion is needed, particularly in a high-risk population. This is to ensure that GC is diagnosed in a timely manner, preferably in its early stages. Patients may benefit from treatment with minimally invasive endoscopic procedures, with the potential of preserving a significant part of the stomach giving the patient a better quality of life [20].

It has been found that the majority of the GC patients, both males and females, were from metropolitan counties (87.4%) with a median household income below the 50^th^ percentile. Karimi et al., in their study, mentioned that low socioeconomic status is a confirmed risk factor for GC. Higher rates of *H. pylori* infection, higher intake of starchy food, or lower access to fresh food and vegetables may be responsible for the association between low socioeconomic status and higher risk of GC [11,21]. Another important finding from our study was that patients from micropolitan counties were at 1.5 times increased risk of mortality, despite lower prevalence compared to metropolitan counties. This can be attributed to relatively lower access to the healthcare system.

There were several limitations which were present in this study. In spite of the fact that NIS is an administrative database, it lacks patient-level information, which could lead to a higher possibility of selection bias. Since our patients were selected from the inpatient setting, there could be a difference in the prevalence of comorbidities when they are compared with the general population. Also, these only included acute inpatients, and there was no further information about if the patient received palliative/comfort care. The comorbidities were included as a binomial variable, and the Charleston comorbidity index was not used after the change in diagnostic coding in the NIS (2019). The re-hospitalizations of patients were not interpreted in this study which would have added to the total inpatient visits. Strength includes that most of the information gathered was from the patient records, which significantly decreases the chances of recall bias. Despite having several limitations, our study provided unique population-based differences in demographics and hospital outcomes in gastric cancer.

## Conclusions

To conclude, females in the age group 50-59 years are at greater risk of all-cause mortality due to GC. Black males are at increased risk of inpatient mortality compared to White males. Significantly increased mortality was seen in females residing in micropolitan counties. Higher all-cause mortality risk was seen in both sexes diagnosed with metastatic GC. And comorbid AIDS increased all-cause inpatient mortality in males, and lymphoma increased all-cause inpatient mortality in females. GC incidence and mortality rates have been down trending with better treatment options, but it still continues to be a major burden on the healthcare system.
